# Lies Kill, Facts Save: Detecting COVID-19 Misinformation in Twitter

**DOI:** 10.1109/ACCESS.2020.3019600

**Published:** 2020-08-26

**Authors:** Mabrook S. Al-Rakhami, Atif M. Al-Amri

**Affiliations:** 1 Research Chair of Pervasive and Mobile ComputingKing Saud University 37850 Riyadh 11543 Saudi Arabia; 2 Information Systems DepartmentCollege of Computer and Information SciencesKing Saud University 37850 Riyadh 11543 Saudi Arabia; 3 Software Engineering DepartmentCollege of Computer and Information SciencesKing Saud University 37850 Riyadh 11543 Saudi Arabia

**Keywords:** Classification, COVID-19, machine learning, misinformation, Twitter

## Abstract

Online social networks (ONSs) such as Twitter have grown to be very useful tools for the dissemination of information. However, they have also become a fertile ground for the spread of false information, particularly regarding the ongoing coronavirus disease 2019 (COVID-19) pandemic. Best described as an infodemic, there is a great need, now more than ever, for scientific fact-checking and misinformation detection regarding the dangers posed by these tools with regards to COVID-19. In this article, we analyze the credibility of information shared on Twitter pertaining the COVID-19 pandemic. For our analysis, we propose an ensemble-learning-based framework for verifying the credibility of a vast number of tweets. In particular, we carry out analyses of a large dataset of tweets conveying information regarding COVID-19. In our approach, we classify the information into two categories: credible or non-credible. Our classifications of tweet credibility are based on various features, including tweet- and user-level features. We conduct multiple experiments on the collected and labeled dataset. The results obtained with the proposed framework reveal high accuracy in detecting credible and non-credible tweets containing COVID-19 information.

## I. Introduction

In recent months, the spreading rate of coronavirus disease 2019 (COVID-19) has been very high around the world [1]–[3] For most people, it is an unprecedented global pandemic in present times. These sentiments have been echoed by the World Health Organization (WHO) Director-General, Tedros Adhanom Ghebreyesus [4]. During the Munich Security Council held on February 15, 2020, he stated that the world was in a war to fight not only a pandemic, but also an infodemic. Various sources have shown that the spread of COVID-19 has been accelerated by the kinds of information, or indeed misinformation, being communicated [3], [4]. This is because individuals act to protect themselves from contracting the disease based on the information they receive from different sources. An example that illustrates the extent to which false information regarding the pandemic has spread was provided in a report by the International Fact-Checking Network (IFCN). ICFN brings together more than a hundred organizations that essentially do fact-checking. By April 2020, they revealed that over 4000 false claims had been spread regarding the pandemic [7]. Unfortunately, misinformation has a horde of adverse outcomes associated with it: it can increase fear of the pandemic among people [8], and it can mislead people when it comes to correct medical practices, as some may follow wrong advice on how to protect themselves from COVID-19, which could lead to ill health or even death [9], [10].

One of the ways through which misinformation is spread is through online social networks (OSNs) such as Twitter [11]. The spread of false information has been seen for other recent epidemics, such a Ebola [12], yellow fever [13], and Zika [14]. This is undoubtedly an alarming trend, since misinformation may be accepted by many individuals as correct information. To curb this “misinformation challenge”, the WHO established the Mythbusters platform, which attempts to demystify false information regarding the COVID-19 pandemic.

These efforts have been complemented by individual fact-checking organizations that have come together under IFCN to increase the efforts of fighting the current infodemic [15].

**FIGURE 1.**
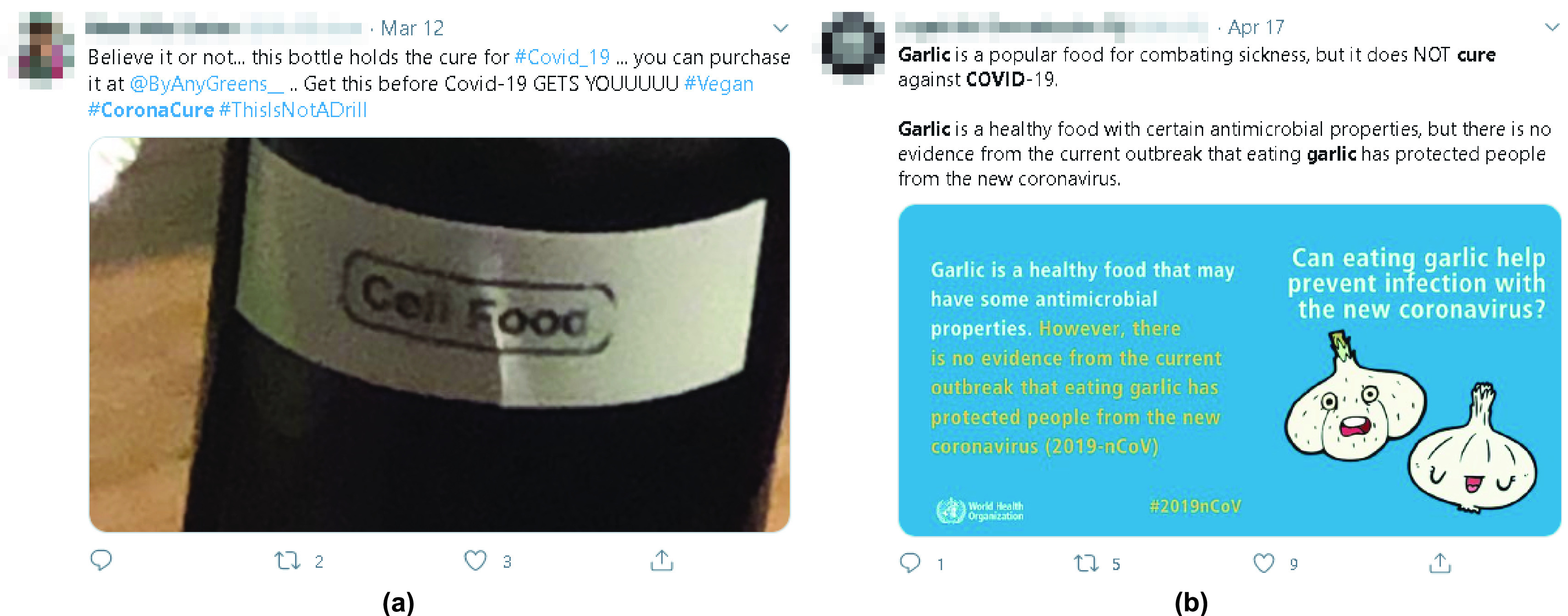
Two example tweets about COVID-19. (a) The left tweet contains misinformation regarding a cure for COVID-19, while (b) the right tweet represents credible information indicating that garlic has not been proved to be a cure against COVID-19.

OSNs have always been associated with the spread of misinformation, where many opinions appear to disregard scientific evidence and material in their posts [16]. A fundamental example that illustrates this is the myriad conversations regarding vaccines and cures to COVID-19. Many people have spread false information regarding COVID-19 cures, and have shown inclination towards accepting it. Figure 1 shows example tweets that promotes false COVID-19 cure and encourages people to purchase it. Another blatant example was the tweet by US President Donald J. Trump that suggested that combining hydroxychloroquine and azithromycin was the “biggest game-changers in the history of medicine” [17]. Medical bodies and medical research institutions subsequently came forth to demystify this false assertion, asking physicians not to follow such information when treating patients. Intended or otherwise, misinformation brings about fear and wrong medical prescriptions. It also leads to defiance of medical guidelines regarding preventive measures, such as social distancing and personal hygiene. This is as the direct result of a horde of misinformation [18].

As a very popular OSN platform, Twitter has become a tool for the spread of misinformation regarding COVID-19 [19], which has resulted in increased mental distress, anxiety, and self-harm. A worrying factor is that the rate at which misinformation is spreading surpasses physical distances, so that the fear of the pandemic spreads alarmingly fast, and seems to be more contagious than the actual COVID-19 [20]. As a result, patients and non-patients are affected both mentally and physically. Therefore, there is the need to investigate and detect misinformation surrounding the fast-moving COVID-19 pandemic, especially through the lens of OSNs.

In response to the current infodemic, this work looks at developing an effective misinformation detection framework with respect to COVID-19, specifically for Twitter. Our major contributions to this area, and the key features of the proposed technique, are summarized as follows:

• We developed a new framework for detecting misinformation on the Twitter platform. In this framework, we integrated six machine-learning algorithms with ensemble learning. Additionally, we enhanced the model by utilizing an ensemble-stacking model with the selected machine-learning models. This resulted in higher accuracy and a more generalized model.
• We collected a large dataset related to the COVID-19 pandemic via Twitter’s streaming application program interface (API) for tweets published from 15 January to 15 April, 2020, aiming to explore the features that constitute the detection of misinformation on Twitter.
• The dataset was assessed and labeled by human annotators. After that, we extracted relevant features related to COVID-19 and applied them when building our framework to automatically measure the credibility of the considered tweets.

The rest of this article is organized as follows, Section II presents some works related to misinformation, and its spread, on OSNs. In Section III, we describe our methodology and framework. Section IV presents the results and a performance evaluation. Section V provides more discussions on the results, the learned lessons and the open directions. Finally, we conclude our work in Section VI.

## II. Related Work

Misinformation can be defined as inaccurate and false information that is spread knowingly or otherwise [21]. The spread of false information has been occasioned by the improved communication technology that allows many people to share information at an affordable cost [22]. Spreading misinformation can have a negative impact on individual human beings and the societies in which they live; it can instill fear in people and, by and large, influence their behavioral responses to such things as elections and natural disasters [23]. One such example is the startling statistics that revealed a high prevalence of fake news being spread in the United States, during the months leading up to the 2016 US elections [20], [21]. Another example is the spread of inaccurate information regarding vaccinations, which has been shown to induce phobia in many parents. As a result, some parents with young children have defied medical guidelines and refused to vaccinate or immunize their children [26]. This has resulted in an unprecedented increase in diseases which could otherwise be prevented.

At the societal level, misinformation was ranked by the World Economic Forum in 2013 as a factor that affects financial status and relationships between various countries [27]. The spread of misinformation has risen to greater levels, so that researchers and scholars are taking a keen interest in understanding how misinformation is spread. They have also sought to discover the driving force that motivates people to share false information regarding various events such as the COVID-19 pandemic or political scenarios [28]. For example, Oh *et al.* [29] carried out an analysis of information obtained from Twitter regarding the Haiti earthquake in 2010. They found out that uncertainty and anxiety with regards to information are fundamental factors that determine how false information spreads. They noted that the best way to counter the spread of misinformation is to cite credible information from reliable sources. Domenico *et al.* [30] also sought to establish how scientific misinformation spreads. They concluded that most people deemed information as credible when their friends tweeted about it often.

In [31], the authors investigated the extraction of information from unstructured text in real-time, which is a challenging process. Their work was based on an embedding method for information retrieval from unstructured text corpora. Focusing on the 2014 Ebola and 2016 Zika outbreaks as use cases, the authors collected related data from Twitter and scholarly abstracts from PubMed. Their study validated the robustness of domain-specific word vector models by comparing them against pre-trained generic word vectors. In another work [32], the authors proposed four metrics to obtain a quantitative assessment of the current maturity of social network analysis technologies: pattern & knowledge discovery, information fusion & integration, scalability, and visualization.

More recent research on the spread of fake news was conducted by Vosoughi *et al.* [33], who revealed that misinformation on Twitter was more prevalent than credible news. This phenomenon was associated with the fact that false news contains unfiltered emotions such as fear or even disgust, to which many people can relate, prompting them to share the news with their Twitter friends. Another possible explanation is that people are fond of sharing new information [33]. Zhao *et al.* [34] conducted a study on how misinformation is spread in the Chinese media. They concluded that when a crisis affecting many people occurs, there is a lot of posting and reposting of misinformation on social media. To counter the spread of fake news, the study determined that the acceptable norms of people and their attitude towards sharing fake news were key factors.

In most previous studies that explored the nature and spread of misinformation on social media [35], the focus was on political news and scientific misinformation. However, t here have been very few studies that focused on the spread of misinformation related specifically to COVID-19, which makes it a critical niche to explore.

## III. Methodology

**FIGURE 2.**
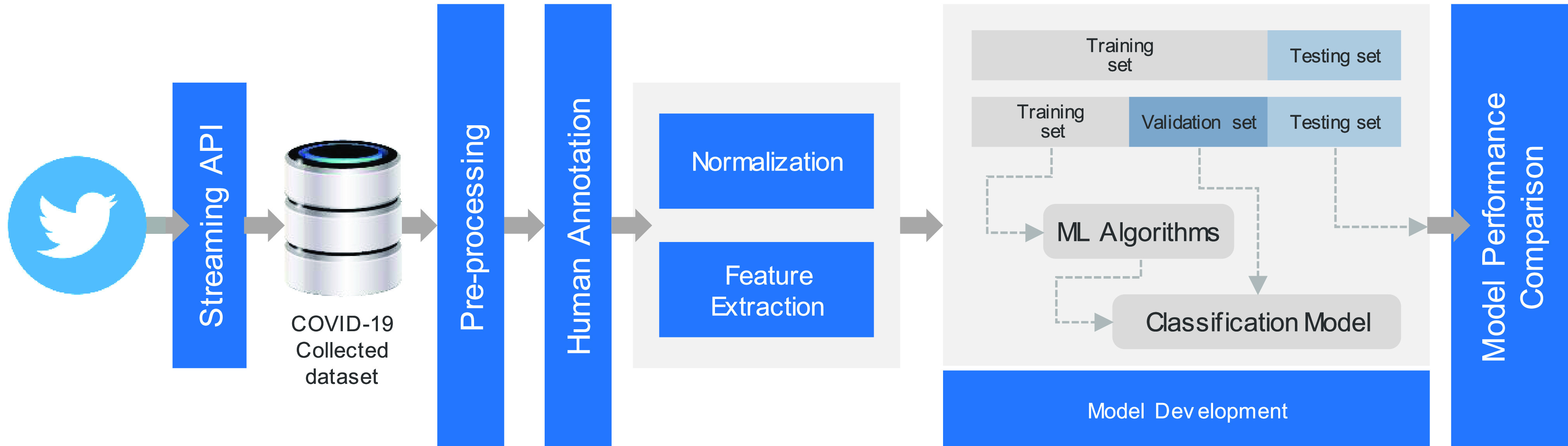
System architecture.

Figure 2 illustrates the system architecture of the proposed framework, starting from collecting the raw data through the use of Twitter’s streaming API, towards performance evaluation and comparison. We modelled the COVID-19 misinformation problem as a binary classification problem. Our method is based on a large dataset which was collected and annotated by human experts. The assumption of the methods used in this work is that each tweet used has specific features. In the following subsections, we describe our steps for collecting and labeling the COVID-19-related data from Twitter. Then, we describe the different feature categories used to assess the credibility of each tweet’s content.

### A. Data Collection

To conduct out experiment, we created a dataset of tweets that was collected using Twitter’s streaming API spanning three months, from 15 January to 15 April, 2020. We used hashtags and keywords related to COVID-19 for our search. Table 1 presents a list of all the relevant keywords used to collect tweets about COVID-19. Identification, annotation, and classification of the tweets were conducted based on the tweet type. In this work, we define a credible COVID-19 tweet as a verified and relevant tweet containing COVID-19 news, vaccination, medication, ways of protection, etc., that had a confirmed, reliable source such as the WHO, IFCN, a fact-checking platform or an official health agency. The non- credible tweets were defined as a collection of non-confirmed or false tweets about COVID-19 that had spread over Twitter. For tweets collected from news segments, detailed research was carried out to determine whether they contained true information. In the case that our annotators determined that a tweet bore false information, it would automatically fall under the “non-credible” category. Furthermore, to increase the accuracy with which the tweets were categorized, annotators would seek opinions from each other. Ultimately, we compiled a dataset of 980,000 tweets. After deleting duplicated and irrelevant tweets, the final dataset contained more than 400,000 tweets.

**TABLE 1:** COVID-19 Related Keywords

Keyword	No. of Tweets
Covid-19	411,301
Covid19	545,183
Covid_19	132,396
Coronavirus	32,072
Covid	1,439
Corona	23,411

**FIGURE 3.**
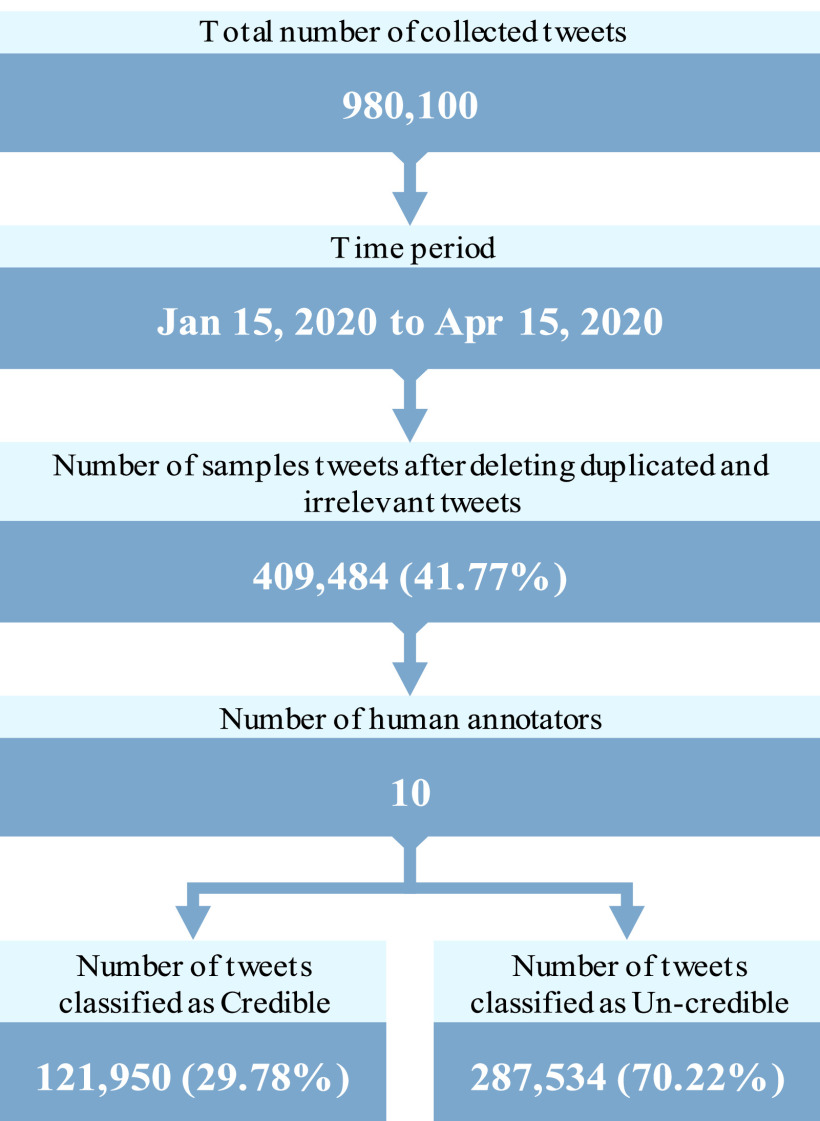
COVID-19 collected data.

Figure 3 illustrates the actual numbers of collected tweets. These tweets were annotated and classified into 121,950 credible tweets and 287,534 non-credible tweets. This dataset forms the primary basis for the subsequent experiments in our work.

### B. Data Annotation and Reliability

The created dataset used in our work was manually annotated by ten human annotators. All the annotators were graduate students with computer and information science majors. Two of the annotators were Ph.D. students, four were master’s degree students and the remaining were bachelor’s degree students. All the annotators were familiar with the annotation process of OSN content. The annotators agreed to perform with the highest level of accuracy during the annotation process. They worked on a part-time basis, and got paid for three months of work. The average count of annotated tweets per day was about 450 tweets per annotator. Along with the collected tweets regarding COVID-19, we conducted a brief initial meeting with the team to describe the desired dataset, gave annotation guidelines, and provided sources from where the annotators could investigate the credibility of each tweet.

To ensure that the annotators would not be biased towards accounts with high favorite and retweet ratios, we passed the dataset instances to our annotators where the visible features of the users’ profiles were omitted. This was done because it is believed that Twitter users with the most followers will likely refrain from spreading misinformation [36].

To evaluate the degree of agreement in the annotation process, and check for inter-agreement between the annotators’ classifications, we constructed a small dataset of 20 tweets, and then invited the ten annotators to decide whether the selected tweets were credible or non-credible. We then applied a Bayesian generalized linear mixed model (GLMM) to their results, which is well-suited for measuring agreement between multiple annotators of binary categorical outcomes [37]. The measure of agreement was 0.74, which indicates good reliability. We believe that this strong chance-corrected agreement was due to the clear guidelines of the annotation process and the credible sources provided for checking the credibility of the COVID-19 tweets under study.

### C. Feature Extraction

The proposed architecture leverages 26 hand-crafted and generic features (described in Table 2) that can be categorized as “tweet-level” and “user-level”. Early work on misinformation detection employed supervised-learning techniques, which have been extensively employed to study manually curated features related to content, users, and networks to seek distinguishing features of online credibility [38]. These studies have shown that those features have the potential for distinguishing between credible and non-credible information.

**TABLE 2:** Description of Extracted Features

Tweet-level features	
${\mathrm {NoRT(}u}_{i})$	No. of retweets
${\mathrm {NoUFav(}u}_{i})$	No. of favorites
$\mathrm {is}{\mathrm {QM(}t}_{i})$	Whether the tweet includes a question mark
$\mathrm {is}{\mathrm {DUP(}t}_{i})$	Whether the tweet is a duplicate of its source
$\mathrm {is}{\mathrm {URL(}t}_{i})$	Whether the tweet contains a URL
${\mathrm {NoURL(}t}_{i})$	No. of URLs embedded in the tweet
${\mathrm {NoMen(}t}_{i})$	No. of mentions
${\mathrm {NoHash(}t}_{i})$	No. of hashtags
${\mathrm {NoWSN(}t}_{i})$	No. of words in the tweet except the source author’s screen name
User-level features	
${\mathrm {NoTwt(}u}_{i})$	No. of tweets user has posted
${\mathrm {NoPL(}u}_{i})$	No. of public lists user belongs to
${\mathrm {NoFlw(}u}_{i})$	No. of followers
${\mathrm {NoFol(}u}_{i})$	No. of followings
$\mathrm {is}{\mathrm {BG(}u}_{i})$	Whether the user profile has a background image
${\mathrm {Rep(}u}_{i})$	User’s reputation (i.e., followers/(followings+1))
${\mathrm {NoTLike(}u}_{i})$	No. of tweets user has liked so far
${\mathrm {Age(}u}_{i})$	Account age in days
${\mathrm {IsV(}u}_{i})$	Whether the profile is verified
${\mathrm {Engage(}u}_{i})$	User engagement (i.e., # posts / (account age+1))
${\mathrm {FlwR(}u}_{i})$	Following rate (i.e., followings / (account age+1))
${\mathrm {FavR(}u}_{i})$	Favorite rate (i.e., user favorites / (account age+1))
${\mathrm {IsGeo(}u}_{i})$	Whether geolocation is enabled
${\mathrm {IsDesc(}u}_{i})$	Whether the user has a description
${\mathrm {NoWDesc(}u}_{i})$	No. of words in the user’s description
${\mathrm {NoCharN(}u}_{i})$	No. of characters in the user’s name including white space
${\mathrm {IsSrc(}u}_{i})$	Whether the user is the source tweet’s author

#### 1) User-Level Features

User-level features represent those features related to the user profile such as number of tweets, number of followers, number of following among others. It is important to mention that some of these features are latent and some are explicitly revealed. For example, the favorite rate is a latent feature, while the number followers is revealed explicitly in the user profile. These features point to the content of the tweet, the user, and the networks. Some works [34]–[36] proved that tweet features curated manually have the capacity to distinguish between credible and non-credible tweets.

#### 2) Tweet-Level Features

In our manual analysis of tweets, we placed a keen focus on content features. We looked at tweets with URLs and embedded multimedia. The purpose of URL usage by Twitter users is to utilize the limited tweeting space [40]. Researchers have shown that many users are likely to share content that has a URL more widely than tweets without [42]. URLs also tend to increase the credibility of the tweet [43]. Notably, it was revealed that tweets that contain URLs go viral more often than ones that do not [44].

### D. Ensemble-Learning-Based Model

Ensemble learning primarily involves training a group of many single machine-learning models (i.e. weak-learners), and then the results are put together with an ensemble scheme [45]. This enhances the ability of the model to generalize, and also enhances the accuracy levels. Accordingly, we selected a model that had the highest performance among the available models for machine learning.

We keenly analyzed each model based on our dataset and enhanced its parameter tuning. We selected four categories of six models to represent weak-learners in addition to comparing their performances. The four categories include: (1) Naive Bayes (NB) and Bayes net (BN) models, (2) a k-nearest neighbor (kNN) model, (3) a decision Tree (DT) that mainly consisted of C4.5 and random-forest (RF) models, and (4) a support vector machine (SVM). We determined the ability of each of the four categories to detect misinformation, after which we selected a set that included weak-learners in addition to meta-models for ensemble learning.

**FIGURE 4.**
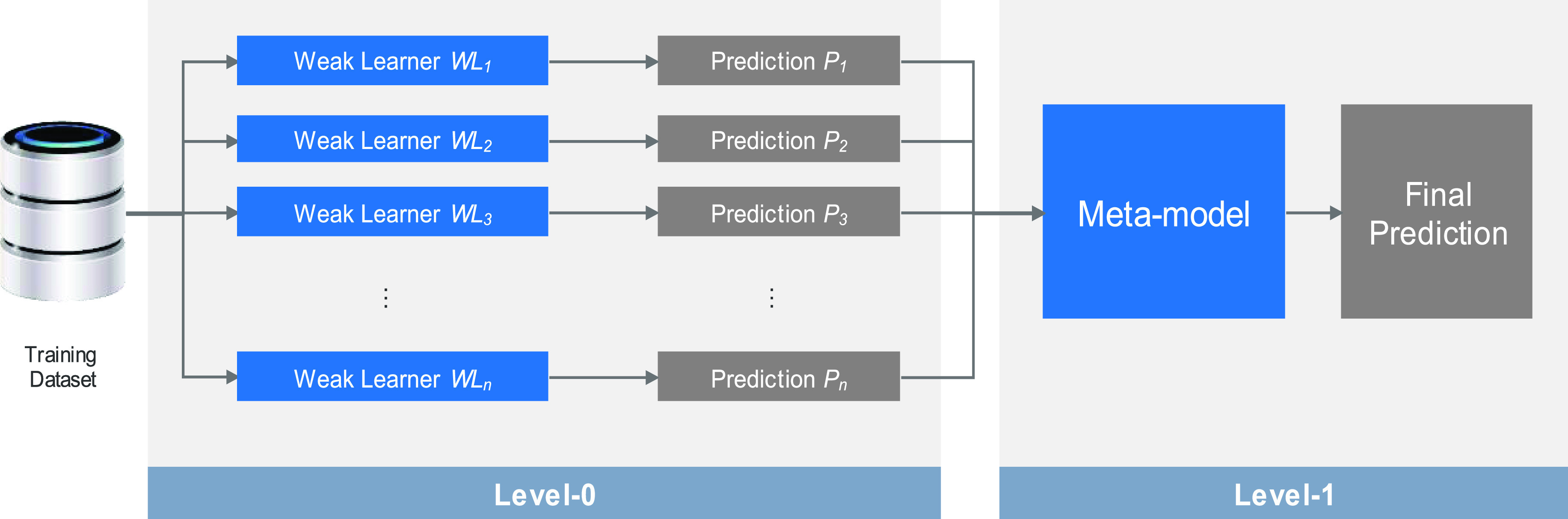
Stacking-based ensemble learning with a two-level structure.

Ensemble learning exists in multiples strategies. The first is a voting-based model [45]. Here, the model calculates the results of diverse weak-learners, after which it picks the set with the most votes. For a bagging-based model [45], sets of weak-learners get the same weights. Second, for boosting models, the learners have weights that are trained based on data which are misclassified, which is done over a sequence of trained models. As illustrated in Fig. 4, a two-level stacking model was constructed using diverse single machine-learning models.

In this work, we employed commonly used machine-learning techniques to create a stacking-based ensemble-learning model. We carried out various experiments when constructing the ensemble model. For a level-0 weak-learner, we used the SVM+RF models, while for a level-1 weak-learner, we used the C4.5 model as a meta-model. We applied $n$-fold cross-validation according to the correlation, entropy, and relief when tuning the ultimate model to attain enhanced performance.

## IV. Experimental Results

### A. Evaluation of Single Weak-Learners

We tested six machine-learning models as candidate weak-learners during the experimental phase: the RF, C4.5, Naive Bayes, Bayes net, SVM and kNN models. Each model was trained based on a 10-fold cross-validation model, which split the overall set into 10 equal subsets. Each category was allocated nine subsets for training and one subset for testing.

**FIGURE 5.**
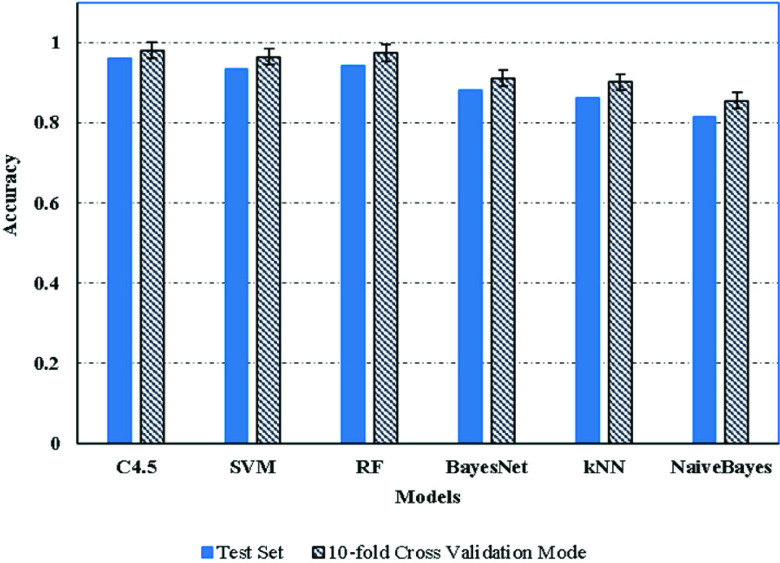
Performance comparison of the base models.

A cross-validation model uses the same overall set for training and testing. We took the average accuracy of each model, as well as the standard deviation, which was used as the 1-sigma error, as shown in Fig. 5. It is clear that C4.5 outperformed all the other models. The average accuracy of the cross-validation model for C4.5 was up to 98%, followed by RF, which had an average accuracy of 97.4%. The Naive Bayes algorithm had the worst performance, with an average accuracy of 85.5%. The results of the average accuracies given for both the cross-validation model and the test set are illustrated in Fig. 5. The standard deviation (error bar) indicates the stability of the selected models.

The models of the test set showed a slight drop in their accuracies compared with the cross validation model, as seen in Fig. 5. For example, the accuracies of kNN and Naïve Bayes drop significantly (by 4.2 % and 4.05%, respectively), despite the fact they performed the best during the training round. This suggests that these models have an over-fitting issue when training sets are involved. The accuracy of C4.5 only dropped by 2%, and therefore it achieved the highest level of accuracy.

In summary, it was established that a single machine-learning model is likely to be the least efficient when applied to new datasets. This was partly ascertained via the applied learning scheme, and also the quality of the used dataset.

### B. Stacking-Based Model

In the preceding section, we analyzed the results from the selected single machine-learning models. It was noted that all models underwent a performance degradation when subjected to the test set. We acknowledge that ensemble learning can possibly transform a single machine-learning model into a robust scheme by integrating several machine-learning models together [40], [41]. However, a fundamental issue of ensemble learning is interpretability losses [46]. Additionally, large models were found to have a vast quantity of parameters in place [47], thus increasing the overfitting problem. A stacking scheme is hence necessary to come up with an acceptable size, which also affects the performance of the given ensemble model and guides the ensemble process. Therefore, we combined models such as level-0 models when performing the stacking. We also replaced the voting module with a meta-model. In this step, the stacking model was used to train the meta-model based on the given available values of the level-0 models.

### C. Selecting the Appropriate Meta-Model

The stacking model design and the workflow are illustrated in Fig. 4. In this model, a secondary dataset was generated for the meta-model training by use of $n$-fold cross-validation. Specially, we used 10-fold cross-validation.

The outcome of the meta-data is critical in the final results. We chose to use the selected meta (level-1) models from C4.5, SVM, and kNN. Our choice was made by considering their stable performance when processing new input, as illustrated in Fig. 5, where each of the six given models was applied as a level-0 model. This was done to measure the performance of diverse models combination. Here, the training and testing sets were similar to the ones used in the test experiments presented in Fig. 5. Table 3 presents the results of this particular experiment, which indicate that the performance of three stacked models has a higher average accuracy than the best single-base model when applied to the new test sets.

**TABLE 3:** Meta-models: Overall Performance

Measurements	Meta-model		
	C4.5	SVM	kNN
Average Accuracy	95.11%	94.67%	94.39%
Kappa Statistic	0.946	0.939	0.937
RRSE	27.34%	30.72%	31.64%
Weighted Precision	0.953	0.947	0.943
Weighted Recall	0.951	0.946	0.943
Weighted F-score	0.951	0.946	0.943
Weighted AUROC	0.972	0.962	0.963

The kappa statistic [48] was also considered alongside the average accuracy, which is often used to measure the degree of agreement between observed and predicted values for a given dataset. The kappa statistic is defined in Eq. 1, where $p$ is the accuracy of the selected model, and $p_{r}$ is the accuracy of the random classifier:

\begin{equation*} k=\frac {p-p_{r}}{1-p_{r}}\tag{1}\end{equation*}

The kappa statistic has been suggested to be a better measure than the simple accuracy. It has also been established that a higher kappa statistic indicates higher accuracy [49]. The root relative squared error (RRSE) was also considered here, which is presented in Eq. 2, where $p_{i}$ is the value predicted by the trained model in the $i$th class (i.e., credible or non-credible), $a_{i}$ is the actual target rate of the $i$th class, and $\bar {a}$ is the mean of the training set. In this work, we calculated the total squared error, which we then normalized by dividing it by the overall squared error of the training set.

\begin{equation*} \mathrm {RRSE}=\sqrt {\frac {{(p_{r}-a_{1})}^{2}\mathrm {+\ldots +}{(p_{n}-a_{n})}^{2}}{{(a_{1}-\bar {a})}^{2}\mathrm {+\ldots +}{(a_{n}-\bar {a})}^{2}}}\tag{2}\end{equation*}

The true positive (TP), true negative (TN) and false negative (FN) are used to calculate the model precision, recall and F-score based on the following equations:

\begin{align*} \mathrm {Precision}=&\frac {\mathrm {TP}}{\mathrm {(TP+TN)}} \tag{3}\\ \mathrm {Recall}=&\frac {\mathrm {TP}}{\mathrm {(TP+FN)}} \tag{4}\\ \mathrm {F-score}=&\frac {\mathrm {2\times Percision\times Recall}}{\mathrm {Percision+Recall}}\tag{5}\end{align*}

The weighted receiver operating characteristic (ROC) area is a probability curve, commonly known as the area under the ROC (AUROC) curve. AUROC uses the weighted TP rate and FP rate values. As shown in Table 4, when applying the SVM+RF combination, the ensemble model achieved better results for all the given measurements.

**TABLE 4:** Ensemble-Models: Overall Performance

	Accuracy (%)	Kappa Statistic (%)	RRSE (%)	Weighted AUROC (%)
C4.5+RF	0.973	0.965	0.378	0.994
SVM+RF	0.978	0.975	0.257	0.997
C4.5+kNN	0.960	0.948	0.336	0.990
SVM+kNN	0.971	0.962	0.297	0.993
SVM+BayesNet +kNN	0.960	0.948	0.319	0.999
C4.5+Bayes Net+kNN	0.960	0.948	0.328	0.996
All models	0.973	0.965	0.288	1.001

### D. Selection of Weak-Learners

As mentioned earlier, one of our aims was to lower the complexity of the model. As such, we attempted to ascertain the smallest set of base models needed to link all models with high accuracy, while at the same time retaining a simple structure. C4.5 was chosen as the preferred meta-model in the previous subsection, and the following seven combinations of base models were considered: C4.5+RF, SVM+RF, C4.5+kNN, SVM+kNN, SVM+BayesNet+kNN, C4.5+Bayes Net+kNN, and finally all the models together. We used the 10-fold cross-validation model as the stacking model. Our aim was to create training and test sets using the initial training set for a given meta-model, and also to test the complete ensemble model using the test set.

**FIGURE 6.**
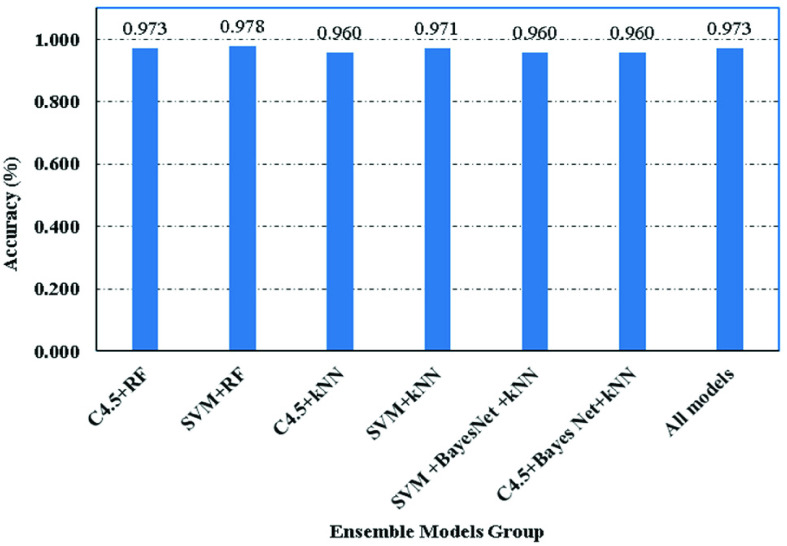
Ensemble-models accuracy comparison.

Figure 6 shows the accuracies of the seven different combinations of base models specified above, and Table 4 shows the performance of each model combination. It is clear that the SVM+RF combination has better accuracy (97.8%) than the C4.5 model (96%), although it has lower performance in terms of the remaining three measurements. The SVM+RF combination had greater accuracy compared to the All model. This indicates that combining many models when creating an ensemble model is not necessary as the weak ones may reduce the overall performance. Consequently, a wisely selected meta-model with appropriate combinations of smaller base models may perform better than all the base models put together.

### E. Selecting the Top Features

**FIGURE 7.**
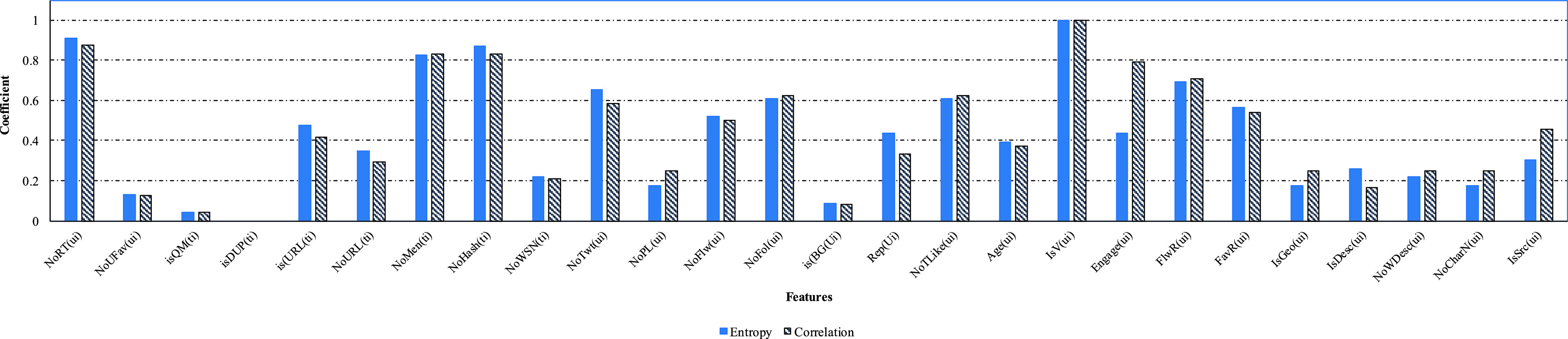
Entropy and correlation ranking for the used features.

To study the effect of feature importance on model prediction, and given that we have in total 26 features (nine tweet-level features and 17 user-level features), we applied two ranking techniques: entropy-based ranking and correlation-based ranking. Entropy-based ranking was applied in the calculations of the acquired symmetrical data, while correlation-based ranking was applied to sort the features needed depending on their correlation with a given class. Figure 7 represents the calculated values for each of the 26 level features. It can be noted that the top five ranked features were sorted as following: ${\mathrm {IsV}(u}_{i})$, ${\mathrm {NoRT}(u}_{i})$, ${\mathrm {NoHash}(t}_{i})$, ${\mathrm {NoMen}(t}_{i})$, and ${\mathrm {FlwR}(u}_{i})$. We can conclude that whether an account is verified or not is the most important feature, followed by the number of retweets, number of hashtags, number of mentions and finally, the following rate.

## V. Discussion

In this section, we discuss some lessons which we have learned through conducting this work. Then, we provide a list of open questions. Finally, we discuss some final thoughts that we like to share with the readers during this ongoing crisis.

### A. Learned Lessons

Conducting this research revealed several critical issues regarding the use of Twitter data when monitoring the problem of the spread of misinformation during an ongoing crisis. We hope to instigate a discussion of this topic with members of the scientific community. First, we realized that the Twitter API limits the range within which the propagation of misinformation can be researched. Besides, Twitter API does not allow for the quick retrieval of data. Since it takes a while before fact-checking organizations prove or deny a given information, it becomes a challenge to find the public’s initial reaction to a given tweet. We also noticed that Twitter opens up COVID-29 data for research purposes [73]. This move, however, does not solve the problem of data retrieval limit.

### B. Open Directions

The spread of misinformation via social media is nothing new, but the current COVID-19 crisis has highlighted the dangers OSNs pose to humanity. This is why there is still a lot of work that needs to be completed regarding the strategies and methods needed to tackle this growing problem. Indeed, the results found here inevitably lead to some key research questions that need to be addressed in the future. We have highlighted four main open research directions in the following.

For starters, there is the need to improve the tools and theories pertaining to social media analytics. This can come in the form of a partially or fully automated way of assessing misinformation. This includes finding the sources of misinformation, the network(s) in which it spreads, how it spreads, how and when it is identified, and the effects such information can have.

Furthermore, since we expect a holistic way of tackling this problem, it is also essential to understand how the results of our work and others in the future can be implemented on other social media platforms, in addition to Twitter. The Twitter network model makes it prone to the spread of misinformation, but other OSNs should also be studied. Indeed, the spread of misinformation regarding COVID-19 is not necessarily restricted to Twitter alone, as it can spread from one platform to another. It is common to see fact-checking platforms failing to refer to any tweets, even in cases where claims are also on Twitter. This is especially the case where the origin of a given claim was on another digital platform, but the sources can be traced back to Twitter, where people might have embedded links from the former OSN. Perhaps a facet that would make an interesting study, in this case, would be closed groups and messages.

The social impact of fake news and misinformation is also an area that requires more research work. Society in general is impacted negatively by the spread of fake news, and studies ought to be conducted to decipher the extent of the impact. We ought to answer questions such as to what extent do the effects occur? Whom do they affect? How can the spread of misinformation be controlled offline? It is also essential to more deeply investigate how the spread of misinformation specifically regarding the COVID-19 pandemic differs from place to place.

Last but not least, there is the need to research this problem in the realms of both social media analytics and crisis management. In this case, social media analytics could borrow from the expertise of managers and researchers in crisis management. This can help them improve their findings and fine-tune their research work. On the flip side, experts in crisis management can use new findings on the propagation of misinformation along with existing models to provide holistic approaches to the way information spreads in a crisis.

### C. Final Thoughts

The spread of misinformation has grown to be a prevalent vice on the Internet over the years. The COVID-19 outbreak has yet again accentuated the risks that come with this growing issue. OSNs have, in particular, been adopted as a way to connect and keep tabs on the information pertaining to the pandemic. However, the spread of misinformation continues to be a major problem as the world continues to grapple with the latest pandemic.

Major international bodies like the WHO and UNESCO are vehemently trying to fight the spread of misinformation related to the COVID-19 outbreak. For example, the WHO has allotted a section of their coronavirus web platform Mythbusters in a bid to tackle fake news. We are, however, in an era of fast-moving information. This begs the question, how can we fight the spread of misinformation and fake news? Well, finding a solution is undoubtedly easier said than done. We are dealing with an infodemic that has forced top organizations and governments to come up with policies that can curb the spread of fake news and misinformation.

A lot of work towards fighting misinformation has come from academia, but OSNs have also joined in the effort. Top social media platforms have crafted tools, systems, and policies that aim at tackling this issue. However, most have yet to find a tangible solution. Platforms like Twitter and Instagram have had to make changes to handle COVID-19 misinformation among users. Many other OSNs have resorted to giving their own verified updates as a way to deal with this problem. Facebook has also shifted its stance on fake news and misinformation by creating new tools and making policy changes. As per these new policies, the term “harmful information” refers to any sort of misinformation that is likely to lead to what is referred to as “imminent physical harm”. This policy is a response to issues like the spread of messages regarding false cures, and false information regarding things like social distancing, etc.

In such a time when the world is facing a pandemic, it is expected that people could be more responsible with the information they spread. Now is the time where citizens have to adhere to government policies rather than spread unnecessary panic, either directly or even indirectly. Unverified information pertaining to COVID-19 is making the situation worse, and we suggest the following measures as a response to this problem:

1) Users should try and cross-check the information they receive to ascertain credibility. They should check and verify the sources: ideally they should source information from reputable digital platforms like the WHO and other channels.
2) In cases were one may encounter any sort of misinformation, then the best way to control the spread is by avoiding any sort of engagement. In this regard, users should refrain from commenting and sharing such content. This is the only way to stop their growth of popularity.
3) If any form of misinformation is shared on social media, then it is also good to report the content. In this case, if the information was shared privately, you can get in touch with the sender and inform them that content shared is likely misinformation. You can explain the risks associated with such content amidst such a global crisis.
4) One can also contribute to sharing credible content from reputable sources on the web. Use sites and platforms that are known to be credible in relaying information regarding COVID-19.

## VI. Conclusion

The ongoing COVID-19 pandemic is a threat to human beings. Unlike other global challenges, such as global warming, containing and defeating COVID-19 will depend much on the quality and credibility of information shared amongst people. However, research has shown that misinformation has spread rapidly on OSNs regarding the pandemic.

In this work, we conducted a comprehensive experiment using real data from the Twitter social network. The results revealed that the proposed ensemble-learning model had better performance than single machine-learning-based models. We enhanced the performance of our stacking model by assessing meta-models and weak-learners. We concluded that the final model size can contain fewer features, and it performed slightly better than the original model. As of now, our model has been designed to detect two categories of tweet credibility: credible or non-credible. We acknowledge that there is room for improvement, part of which shall include the following. First is the incorporation of complex tweets containing news and emotional content. Additionally, we shall take into consideration other OSNs that will help us enhance our dataset. Secondly, we shall look out for updated ensemble techniques and machine-learning methods to enhance the current model.
